# Evaluation of sustainable and healthy eating behaviors and adherence to the planetary health diet index in Turkish adults: a cross-sectional study

**DOI:** 10.3389/fnut.2023.1180880

**Published:** 2023-10-02

**Authors:** Melahat Sedanur Macit-Çelebi, Osman Bozkurt, Betul Kocaadam-Bozkurt, Eda Köksal

**Affiliations:** ^1^Department of Nutrition and Dietetics, Ondokuz Mayıs University, Samsun, Türkiye; ^2^Department of Nutrition and Dietetics, Erzurum Technical University, Erzurum, Türkiye; ^3^Department of Nutrition and Dietetics, Gazi University, Ankara, Türkiye

**Keywords:** planetary health diet, sustainable diets, obesity, healthy eating behaviors, sustainability

## Abstract

**Aim:**

The Planetary Health Diet Index (PHDI) is a relatively new index, and studies about its relationship with eating behaviors, nutritional status, and obesity in adults are very limited. For this reason, in this study, sustainable healthy eating behaviors of individuals and compliance of their diets with PHDI were evaluated.

**Methods:**

This cross-sectional study was conducted with 1,112 adults (70.1% women and 29.9% men with mean age = 28.7 years, SE = 9.47). Study data were obtained with the face-to-face interview method via a questionnaire including sociodemographic characteristics, anthropometric measurements, the Sustainable and Healthy Eating (SHE) Behaviors Scale, and 24-h dietary recall. PHDI was evaluated for adherence to EAT-Lancet Commission recommendations.

**Results:**

The average PHDI total score was 41.5 points. Higher SHE Behaviors Scale and PHDI scores were observed in participants with a duration of education above 8 years (*p* < 0.05). Those with lower SHE Behaviors Scale and PHDI scores were more likely to be obese (*p* < 0.001). The total PHDI score was positively associated with fiber, vitamin E, potassium, and folate, and negatively associated with pyridoxine and calcium (*p* < 0.05). The total SHE Behaviors Scale score was positively associated with carbohydrates, fiber, and potassium and negatively associated with pyridoxine, calcium, and energy (*p* < 0.05). A one-unit increase in SHE Behaviors Scale total score resulted in a 5,530 unit (95%CI: 4.652; 6.407) increase in PHDI total score and a one-unit increase in duration of education (years) resulted in a 0.660 unit (95%CI: 0.403; 0.918) increase in PHDI total score. Furthermore, a one-unit increase in Body Mass Index (BMI) (kg/m^2^) resulted in a − 0.218 unit (95%CI: −0.424; −0.013) decrease in PHDI total score.

**Conclusion:**

The participants’ PHDI index scores were low; therefore, the adherence to the EAT-Lancet recommendation was low which might be associated with obesity. Clinical studies evaluating the effects of adherence to sustainable diets on adequate and balanced nutrition and health outcomes are recommended.

## 1. Introduction

The United Nations predicts the world’s population will grow to 9.7 billion in 2050 (1). The Food and Agriculture Organization (FAO) estimates that by 2050, food production must expand by at least 62% to meet the needs of the rising global population (2). In recent years, the development of sustainable food systems has led to changes in traditional agricultural production systems, affecting human diets (3). In addition to these developments and changes, the definition of a healthy diet has been examined and revised to incorporate planetary health principles. According to the Food and Agriculture Organization and the World Health Organization (WHO), sustainable healthy diets are “dietary patterns that promote all aspects of an individual’s health and wellbeing; have low environmental pressure and impact; are accessible, affordable, safe and equitable; and are culturally acceptable” (4). In this line, the EAT-Lancet Commission on “Healthy Diets from Sustainable Food Systems” (EAT-Lancet) suggested the “Planetary Health Diet.” This reference diet is based on the food system’s environmental and human health effects. These guidelines are built on a diet rich in vegetables, greens, fruits, and whole grains and low in meat, fish, eggs, refined cereals, and tubers (5).

Plant-based diets consist of fresh and lightly-cooked foods, and the energy required for plant-based food production is substantially less than for meat preparation. Moreover, plant-based diets result in a smaller environmental footprint, which enhances the quality of soil, water, and air, thereby enhancing the health of all living organisms. Consuming seasonal fruits and vegetables in plant-based diets reduces energy consumption and promotes sustainable resource management (6). In addition, plant-based diets are energy-efficient diets. They have a smaller effect on climate change than animal-based diets (7). In order to meet the rising *per capita* demand of a growing population by 2050, the meat industry would need to increase production by 50–73% (8). However, this situation seems unlikely to be sustained.

According to the EAT-Lancet report, adopting the recommendations for a healthy and sustainable diet might prevent 11 million deaths annually (5). The main goal of the EAT-Lancet diet is to enhance population and environmental health. The report demonstrates that this reference diet is nutritionally balanced and has a low ecological impact (9). Studies evaluating adherence to EAT-Lancet guidelines in various scenarios and countries are very interesting. One study found an inverse relationship between the EAT-Lancet diet score and ischemic heart disease and diabetes (10). In the Swedish population, high adherence to the EAT-Lancet diet was associated with a decreased risk of incident diabetes among people with different genetic risks (11). Based on the EAT-Lancet diet, Cacau et al. recently proposed the Planetary Health Diet Index (PHDI), which consists of 16 components that score proportionally and consider all EAT-Lancet food groups in addition to energetic density (12).

PHDI was associated with higher overall dietary quality and lower greenhouse gas emissions (12, 13). According to a study, the Brazilian population showed low adherence to a healthy and sustainable dietary pattern and seems far from meeting the EAT-Lancet recommendations (14). Furthermore, this study showed that women, the elderly, those who are overweight or obese, and those living in urban areas had higher scores in the PHDI (14). However, another study showed that higher adherence to the PHDI may decrease obesity indicators (9). A recent study showed that obesity may affect the feasibility of a sustainable environment (15).

The PHDI is a relatively new index, and there is limited data on its relationship with eating behavior, nutritional status, and obesity in adults. In this study, sustainable healthy eating behaviors of individuals, compliance of their diets with PHDI, and the factors affecting them were evaluated.

## 2. Methods

The convenience sampling method was used for data collection. The study data were obtained with face-to-face interviews via a questionnaire prepared by the researchers. A total of 1,215 potential participants were reached personally or invited by e-mail to take part in the study at the Erzurum Technical University Department of Nutrition and Dietetics. However, 103 of the participants did not complete the questionnaire, were unwilling or unable to provide informed consent, and had severe acute or chronic diseases. Therefore, this cross-sectional study was conducted with 1,112 adults (70.1% women and 29.9% men with mean age = 28.7 years, SE = 0.34) between September 2022 and February 2023 in Erzurum (one of the metropolitan cities in the east of Turkiye). The inclusion criteria were meeting the age criteria (19–64 years), not having chronic health conditions or psychological disorders, consented to participate, and not following a special diet or eating model. The exclusion criteria were the inability or reluctance to complete the questionnaire, being pregnant or breastfeeding, not meeting the age requirement, and following a special diet or eating model. To carry out this research, “Ethics Committee Approval” was received from the Erzurum Technical University Ethics Committee (number of meetings: 8, decisions: 5, date: 29.08.2022). The research was carried out following the Declaration of Helsinki. Informed consent was obtained from the participants.

The questionnaire included sociodemographic characteristics, anthropometric measurements, the Sustainable and Healthy Eating (SHE) Behaviors Scale, and 24-h dietary recall. The dietary energy and nutrient intakes were evaluated using the Nutrition Information System (BeBiS) program (version 9). Height and body weight measurements were self-reported. By dividing the body weight by the square of the height, the Body Mass Index (BMI) was calculated. Participants with a BMI below 18.50 kg/m^2^ were classified as underweight, with a BMI in the range of 18.50–24.99 kg/m^2^ were classified as normal, with a BMI in the range of 25.0–29.99 kg/m^2^ were classified as overweight, and those with a BMI ≥30.0 kg/m^2^ were classified as obese (16).

### 2.1. Sustainable and healthy eating behaviors scale

The Sustainable and Healthy Eating (SHE) Behaviors Scale was developed by Żakowska-Biemans et al. to assess adults’ self-reported sustainable and healthful eating behaviors (17). Koksal et al. conducted the Turkish adaptation, validity, and reliability study of the scale (18). Cronbach-α values of the scale and its subscales ranged from 0.764 to 0.912. This scale comprises 32 items and 7 factors. The items are rated on a seven-point Likert scale (never to always). The seven factors are quality labels, seasonal food and avoiding food waste, animal welfare, meat reduction, healthy and balanced diet, local food, and low fat. The mean of all the factor values is considered when calculating the overall scale score. Factor scores are calculated by taking the average of the scores (between 1 and 7 points) given to the items in that factor. In calculating the total scale score, the average of the scores given to all factors is taken (score range 1 to 7) (18). Increasing results on both the overall and the subscales indicate an increase in sustainable and healthy eating behaviors (18).

### 2.2. The planetary health diet index

In the present study, 24-h dietary recall (for 1 day) of the participants was taken by the researchers. Energy and nutrient intakes were evaluated using the Nutrition Information System (BeBiS) program (The Food Code and Nutrient Data Base, BLS II.3, 1999, version 9.0).

The Planetary Health Diet Index (PHDI) was developed from the EAT Lancet Commission’s dietary recommendations (12). The daily energy intake for the EAT-Lancet Commission’s dietary recommendations was set at 2,500 kcal. Each component can receive a maximum of 10 or 5 points, resulting in a PHDI score between 0 and 150 (12). PHDI is calculated based on recommendations for various dietary energy intakes and assessed based on how much each food group contributes to the total energy. The PHDI uses a gradual scoring system and is an energetic density index. This diet is based on 16 food components (adequacy component: nuts and peanuts, legumes, fruits, vegetables, and whole cereals; optimum component: eggs, fish and seafood, tubers and potatoes, dairy, and vegetable oils; ratio component: dark green vegetables/total ratio and red vegetables/total ratio; moderation component: red meat, chicken substitutes, animal fats, and added sugars) (12, 13). The calculation of the PHDI is explained in detail in relevant studies (9, 12); please see Supplementary File 1.

### 2.3. Data analysis

The Statistical Package for the Social Sciences (version 23.0) software was used for data analyses. The variables were evaluated using visual (histogram and probability graphs), and skewness and kurtosis (from −1 to 1) to determine whether or not they were normally distributed. Data were evaluated with descriptive statistics such as mean, standard error (SE), number, and percentage. Participants’ SHE Behaviors Scale total and subgroup scores and BMI values (kg/m^2^) were given according to PHDI score quartiles. Chi-square analysis was used to compare qualitative data and detect differences between groups. For comparison, the T-test, the Mann–Whitney U test, One-Way ANOVA, or the Kruskal Wallis test were used in independent groups. For post-doc analysis, Bonferroni correction was applied for multiple pairwise comparisons. The Pearson correlation analysis was used for the relationship between variables.

Furthermore, linear regression was used to determine factors related to the PHDI score to explain the relationships between observable associations. SHE total score, BMI, and duration of education (years) were selected as predictors, and the model was adjusted for age and sex (0 for women and 1 for men). The results were interpreted with 95% confidence.

## 3. Results

Of the total 1,112 participants (mean age = 28.7 years, SE = 9.47) enrolled in the study, 779 (70.1%) were women and 333 (29.9%) were men. More than half of the participants were not working (69.9%) and were single (64.6%). The duration of education of most of the participants (87.0%) was over 8 years (mean duration of education was 13.6 years, SE = 3.60). In this cross-sectional study, obesity indices were also examined. Accordingly, a total of 613 (55.1%) participants (mean BMI = 24.0 kg/m^2^, SE = 4.50) were in the normal BMI (kg/m^2^) group, and 407 (36.6%) were in the overweight/obese group.

In this study, the average PHDI total score was 41.5 points, with a total score that can range from 0 to 150. A descriptive analysis of PHDI components is presented in Supplementary File 2.

In the total of participants, the working (mean = 4.0, SE = 0.05) and married (mean = 3.8, SE = 0.05) groups had higher scores on the SHE Behaviors Scale compared to the not working (mean = 3.6, SE = 0.03, *p* < 0.001) and single group (mean = 3.6, SE = 0.03, *p* < 0.05). Higher scores on the SHE Behaviors Scale and higher PHDI scores were also observed in participants with a duration of education above 8 years (mean = 3.6, SE = 0.02, *p* < 0.001; mean = 43.1, SE = 0.48, *p* = 0.001, respectively). Those with lower SHE Behaviors Scale and PHDI scores were more likely to be obese (*p* < 0.001; Table 1).

**Table 1:** Sustainable and healthy eating (SHE) behaviors scale and planetary healthy eating index (PHDI) total scores of participants according to their sociodemographic characteristics.

	*n* (%)	SHE behaviors scale total score		PHDI total score	p*
		Mean ± SE	*p* ^§^	Mean ± SE	
Sex					
Women	779 (70.1%)	3.7 ± 0.35	0.178	41.6 ± 0.59	0.559
Men	333 (29.9%)	3.6 ± 0.05		41.0 ± 0.80	
Working status					
Yes	335 (30.1%)	4.0 ± 0.05	<0.001**	42.5 ± 0.80	0.126
No	777 (69.9%)	3.6 ± 0.03		40.9 ± 0.57	
Educational duration					
< 8 years	145 (13.0%)	3.2 ± 0.11	<0.001**	30.3 ± 1.46	0.001**
> 8 years	967 (87.0%)	3.6 ± 0.02		43.1 ± 0.48	
Marital status					
Married	394 (35.4%)	3.8 ± 0.05	0.011*	39.2 ± 0.88	0.001*
Single	718 (64.6%)	3.6 ± 0.03		42.7 ± 0.56	
BMI classification					
Underweight	92 (8.3%)	3.6 ± 0.09^a^		41.8 ± 1.59^a^	
Normal	613 (55.1%)	3.8 ± 0.03^a^	<0.001**	43.2 ± 0.61^a^	<0.001**
Overweight	300 (27.0%)	3.8 ± 0.04^a^		41.7 ± 0.83^a^	
Obese	107 (9.6%)	2.9 ± 0.13^b^		30.1 ± 1.98^b^	

^§^Difference between SHE total score according to the groups, *Difference between PHDI total score according to the groups. Data are given as numbers and (*n*) and percent (%). *p < 0.05, ** *p* < 0.001. ^a,b^ Same letters within a column indicate no differences according to pairwise comparisons.

Table 2 presents SHE Behaviors Scale total and subgroup scores, and BMI values (kg/m^2^) according to PHDI quartiles. Those with the highest PHDI quartile (Q4: 51.99–98.96 points) had the highest quality labels score (mean = 3.9, SE = 0.07; *p* < 0.05) and seasonal food score (mean = 4.4, SE = 0.6; *p* < 0.001) compared to Q1 group. The participants in the highest quartile had higher healthy and balanced diet scores (mean = 4.7, SE = 0.07) compared to Q1 (mean = 4.1, SE = 0.07), Q2 (mean = 4.3, SE = 0.07) and Q3 (mean = 4.3, SE = 0.07) quartiles and higher local food scores (mean = 3.3, SE = 0.08) compared to Q1 (mean = 2.9, SE = 0.07) and Q3 (mean = 2.9, SE = 0.07) group (*p* < 0.001). Meat reduction (mean = 3.4, SE = 0.08; *p* < 0.05), animal welfare (mean = 3.9, SE = 0.08; *p* = 0.001), and low fat (mean = 4.9, SE = 0.08; *p* < 0.001) subscores were the highest in the Q4 group. SHE Behaviors Scale total scores were statistically different between all PHDI quartiles (*p* < 0.001). The participants with the highest PHDI scores tended to have the lowest BMI values (mean = 23.5, SE = 0.22 kg/m^2^; *p* < 0.001) compared to Q1 group.

**Table 2:** SHE subgroup scores, SHE total score, and body mass index (BMI) values (kg/m^2^) of participants according to PHDI score quartiles.

	Q1 (*n* = 278) (0–30.56)	Q2 (*n* = 278) (30.56-40.61)	Q3 (*n* = 278) (60.61-51.99)	Q4 (*n* = 278) (51.99-98.96)	*p*
	Mean ± SE	Mean ± SE	Mean ± SE	Mean ± SE	
Quality labels	3.6 ± 0.67^a^	3.6 ± 0.06^ab^	3.6 ± 0.06^b^	3.9 ± 0.07^b^	0.004*
Seasonal food	4.0 ± 0.07^a^	4.2 ± 0.06^ab^	4.2 ± 0.06^ab^	4.4 ± 0.06^b^	0.002*
Healthy & balanced diet	4.1 ± 0.07^a^	4.3 ± 0.07^a^	4.3 ± 0.07^a^	4.7 ± 0.07^b^	<0.001**
Local food	2.9 ± 0.08^a^	3.0 ± 0.07^abc^	2.9 ± 0.07^b^	3.3 ± 0.08^c^	0.001**
Meat reduction	3.0 ± 0.08^a^	3.2 ± 0.07^a^	3.1 ± 0.07^a^	3.4 ± 0.08^b^	0.014*
Animal welfare	3.4 ± 0.08^a^	3.6 ± 0.07^a^	3.7 ± 0.08^a^	3.9 ± 0.08^b^	<0.001**
Low fat	4.4 ± 0.08^a^	4.5 ± 0.08^a^	4.6 ± 0.07^a^	4.9 ± 0.08^b^	0.001**
SHE Behaviors Scale total score	3.2 ± 0.07^a^	3.8 ± 0.05^b^	3.8 ± 0.04^c^	4.1 ± 0.05^d^	<0.001**
BMI (kg/m^2^)	24.9 ± 0.32^a^	23.6 ± 0.26^b^	24.0 ± 0.25^ab^	23.5 ± 0.22^b^	<0.001**

Data are given as mean ± standard error and number (*N*) and percent (%). *p < 0.05, **p < 0.001. ^a, b, c^ Same letters within a row indicate no differences according to pairwise comparisons.

Table 3 presents the correlations between PHDI, SHE Behaviors Scale and subgroups, and BMI. There was a positive correlation between the SHE Behaviors Scale and PHDI (*r* = 0.374, *p* < 0.001). All SHE Behaviors Scale subgroups positively correlated with the PHDI scores (*p* < 0.001). Body mass index values (kg/m^2^) were negatively correlated with PHDI and SHE Behaviors Scale scores (*r* = −0.159, *r* = −0.130, *p* < 0.001, respectively). Furthermore, quality labels and seasonal food and avoiding food waste subscales scores correlated negatively with BMI values (kg/m^2^; *p* < 0.001).

**Table 3:** Correlation (r) of PHDI, SHE behaviors scale, and BMI.

	PHDI total score	SHE behaviors scale	Quality labels	Seasonal food and avoiding food waste	Healthy & balanced diet	Local food	Meat reduction	Animal welfare	Low fat	BMI
PHDI total score										
SHE Behaviors Scale	0.374^**^									
Quality labels	0.118^**^	0.635^**^								
Seasonal food and avoiding food waste	0.125^**^	0.609^**^	0.588^**^							
Healthy & balanced diet	0.158^**^	0.635^**^	0.627^**^	0.570^**^						
Local food	0.108^**^	0.535^**^	0.433^**^	0.371^**^	0.305^**^					
Meat reduction	0.099^**^	0.511^**^	0.338^**^	0.319^**^	0.273^**^	0.390^**^				
Animal welfare	0.118^**^	0.603^**^	0.519^**^	0.470^**^	0.480^**^	0.416^**^	0.347^**^			
Low fat	0.138^**^	0.552^**^	0.433^**^	0.503^**^	0.551^**^	0.216^**^	0.285^**^	0.485^**^		
BMI (kg/m^2^)	−0.159^**^	−0.130^**^	−0.121^**^	−0.156^**^	0.056	−0.026	0.067	0.074	0.062	

*BMI, body mass index; SHE, Sustainable Healthy Eating. *p < 0.05, **p < 0.001.

Table 4 presents the association between PHDI and SHE behaviors scale scores, and nutrients. The total PHDI score showed a positive association with fiber (g), vitamin E (mg), potassium (mg), and folate (μg). It was negatively associated with pyridoxine (mg) and calcium (mg; *p* < 0.05). The total SHE Behaviors Scale score showed a positive association with carbohydrate (g), fiber (g), and potassium (mg) and was negatively associated with pyridoxine (mg), calcium (mg), and energy (kcal; *p* < 0.05).

**Table 4:** Association between PHDI scores and SHE behaviors scale scores and nutrients.

	PHDI scores					SHE behaviors scale				
	*β*	*t*	95% CI		*p*	*β*	*t*	95% CI		*p*
(Constant)		37.073	39.310	43.704	<0.001		50.190	3.521	3.808	<0.001
Energy (kcal)	0.052	0.024	−0.074	0.075	0.981	−4.437	−1.986	−0.010	0.000	**<0.001**
Protein (g)	0.111	0.316	−0.272	0.377	0.752	0.725	1.962	0.000	0.042	0.057
Fat (g)	−0.121	−0.123	−0.699	0.616	0.902	2.035	1.981	0.000	0.085	0.050
Carbohydrate (g)	−0.282	−0.242	−0.340	0.265	0.809	2.448	2.026	0.001	0.040	**0.048**
Fiber (g)	0.213	2.720	0.073	0.451	**0.007**	0.045	0.518	−0.010	0.017	**0.043**
Vitamin A (μg)	0.031	0.460	−0.001	0.001	0.646	−0.047	−0.636	0.000	0.000	0.604
Vitamin D (μg)	0.028	0.862	−0.067	0.172	0.389	−0.005	−0.147	−0.008	0.007	0.525
Vitamin E (mg)	0.111	2.870	0.043	0.229	**0.004**	−0.022	−0.543	−0.008	0.004	0.883
Vitamin K (μg)	0.034	0.966	−0.004	0.012	0.334	0.020	0.544	0.000	0.001	0.587
Thiamine (mg)	0.121	1.424	−1.340	8.436	0.155	0.049	0.562	−0.224	0.404	0.587
Riboflavine (mg)	0.043	0.387	−3.487	5.203	0.698	0.204	1.516	−0.074	0.578	0.575
Niacin (mg)	−0.139	−1.641	−0.308	0.027	0.101	−0.139	−1.518	−0.020	0.003	0.130
Vitamin B_5_ (mg)	−0.120	−1.519	−1.536	0.196	0.129	−0.196	−2.328	−0.126	−0.011	0.129
Pyridoxine (mg)	−0.184	−2.542	−6.240	−0.803	**0.011**	−0.097	−1.257	−0.294	0.064	**0.020**
Vitamin B_12_ (μg)	−0.066	−0.918	−0.287	0.104	0.359	−0.029	−0.377	−0.016	0.011	0.706
Vitamin C (mg)	−0.061	−1.726	−0.017	0.001	0.085	−0.015	−0.402	−0.001	0.000	0.688
Sodium (mg)	−0.002	−0.054	−0.001	0.001	0.957	−0.059	−1.696	0.000	0.000	0.090
Potassium (mg)	0.318	4.395	0.002	0.007	**<0.001**	0.224	2.942	0.000	0.000	**0.003**
Calcium (mg)	−0.142	−2.779	−0.012	−0.002	**0.006**	−0.198	−3.255	−0.001	0.000	**0.001**
Magnesium (mg)	−0.102	−1.055	−0.031	0.009	0.292	−0.155	−1.267	−0.003	0.001	0.205
Iron (mg)	−0.016	−0.198	−0.485	0.396	0.843	0.174	0.967	−0.042	0.016	0.375
Zinc (mg)	−0.007	−0.092	−0.450	0.410	0.927	−0.048	−0.580	−0.036	0.019	0.562
Folate, total (μg)	0.145	2.104	0.001	0.025	**0.036**	0.056	0.781	0.000	0.001	0.405
Phosphorus (mg)	0.206	1.172	−0.003	0.000	0.241	0.174	0.967	0.000	0.001	0.334

The bold values are indicates significant at *p* < 0.05.

A one-unit increase in SHE Behaviors Scale total score resulted in a 5,530 unit (95%CI: 4.652; 6.407) increase in PHDI total score, and a one-unit increase in duration of education (years) resulted in a 0.660 unit (95%CI: 0.403; 0.918) increase in PHDI total score. Furthermore, a one-unit increase in BMI (kg/m^2^) resulted in a − 0.218 unit (95%CI: −0.424; −0.013) decrease in PHDI total score.

## 4. Discussion

To the best of our knowledge, this study is the first to evaluate sustainable healthy eating behaviors of individuals and compliance of their diets with PHDI in Turkish adults. This research showed the participants’ PHDI index scores were low; therefore, the adherence to the EAT-Lancet recommendation was low. High BMI was determined to be associated with low PHDI scores and SHE Behaviors Scale scores. Furthermore, high education level positively related to SHE Behaviors Scale and PHDI scores. Individuals with higher SHE Behaviors Scale scores also had higher PHDI scores. When the diets of the individuals were examined, dietary energy was not found to be associated with the SHE Behaviors Scale score and PHDI score. However, the PHDI score was positively correlated with fiber, vitamin E, potassium, and folate and negatively correlated with calcium and pyridoxine. SHE Behaviors Scale scores were positively correlated with dietary carbohydrate, fiber, and potassium intake but negatively correlated with pyridoxine and calcium intake, similar to the PHDI score.

This research showed that the mean PHDI score is 41.5, corresponding to the Q2 quartile when evaluated according to the PHDI quartile distributions. In a study conducted in Brazil, PHDI index scores were comparable to ours (45.9 points), and population compliance with EAT-Lancet recommendations was low (14). However, in a study conducted by Cacau et al., the mean PHDI score was 60.4 (12). In our research, adherence to EAT-Lancet recommendations was low, depending on the PHDI scores.

According to the World Health Organization (WHO) report, Turkiye reports the highest obesity rate for adults in Europe (32.1%), with the rate in the rest of Europe at 23.3% (19). In this study, 36.6% of the participants were overweight or obese. The increasing prevalence of obesity in our country and globally is alarming, as are its adverse effects on environmental health and sustainability. Unhealthy eating habits that cause obesity do not comply with planetary health principles (20). It is stated that obesity is associated with low sustainable and healthy eating behaviors and low sustainability of diet (9, 15). A study revealed that overweight individuals have low Sustainable Diet Index scores (21). In a study evaluating obesity outcomes of adherence to PHDI, individuals with high adherence to the PHDI had lower BMI (*β* = −0.50; 95%CI: −0.73; −0.27) and waist circumferences (*β* = 1.70; 95%CI: −2.28; −1.12) values (9). However, another study showed that overweight/obese individuals had higher PHDI scores (14). This study determined that high BMI was associated with low PHDI and SHE Behaviors Scale scores. Our regression model concurs with this finding; as a result, a decrease in BMI is associated with an increase in the PHDI total scores (Table 5). Therefore, more studies are needed on the effects of obesity on the sustainable environment and the effects of sustainable diets on obesity prevalence.

**Table 5:** Multiple linear regression model for the prediction of planetary healthy eating index (PHDI) total score.

	PHDI				
Independent variables	B	SE	CI 95%	*p*	Adjusted *R*^2^
SHE total score	5.530	0.447	4.652; 6.407	<0.001**	0.169
Education duration	0.660	0.131	0.403; 0.918	<0.001**	
BMI (kg/m^2^)	−0.218	0.105	−0.424; -0.013	0.030*	

a. Dependent variable: Planetary Healthy Eating Index (PHDI). b. Predictors: (Constant), Sustainable and Healthy Eating (SHE) Behaviors Scale; Body mass index (BMI); education duration (years); Adjusted for: Sex (0-women, 1-men); and age (years), *p < 0.05, **p < 0.001.

Another significant result of our research is a positive relationship between education level, sustainable and healthy eating behaviors, and PHDI scores. Our study showed that a one-unit increase in the duration of education (years) resulted in a 0.660 unit (95%CI: 0.403; 0.918) increase in PHDI total score (*p* < 0.05). A study reported that increased duration of education was positively associated with increased awareness of reducing individual ecological footprint (15). It is crucial to provide education on sustainable and healthy eating behaviors and the environmental effects of diet. In this study, the increase in the scores obtained from the SHE behaviors scale was also positively associated with the increase in the PHDI scores. In line with these results, education is essential for sustainable environmental health (22).

According to a study, higher PHDI scores were associated with higher overall dietary quality and lower greenhouse gas emissions (12). In the results of our study, no relationship was found between dietary energy, protein and PHDI scores. Cacau et al. also found no association between dietary energy, dietary protein, and PHDI scores (12). Another important result in our study concerns dietary fiber. It is noteworthy that dietary fiber positively affects scores regarding nutrients. Dietary fiber draws attention to its significant functionality in non-communicable diseases, especially obesity (23). Sustainable diets are rich in vegetables, greens, fruits, and whole grains and low in meat, fish, eggs, refined cereals, and tubers. For this reason, the results of this research support that sustainable and healthy eating behaviors and high adherence to PHDI increase dietary fiber intake. There is also a concern that sustainable diets may adversely affect the intake levels of some nutrients (iron, retinol, vitamin B12, etc.) due to recommendations to reduce animal-derived food consumption (24). In European adolescents, higher PHDI scores were associated with a greater intake of nutrients predominantly from plant-source foods, such as vegetable protein, vitamin E, and folate, and with a lower intake of nutrients predominantly from animal-source foods (25). In this study, it was determined that daily intake levels of dietary calcium and pyridoxine from foods of primarily animal origin were negatively associated with high SHE Behaviors and PHDI scores, whereas vitamin E, potassium, and folate from foods of mostly plant origin were positively associated with high SHE Behaviors and PHDI scores. Cacau et al. reported similar results for pyridoxine and its association with PHDI scores. They also revealed that carbohydrates, polyunsaturated fats, fiber, vitamins C, A, E, and K, thiamine, folate, iron, phosphorus, potassium, zinc, selenium, magnesium, and copper are positively associated with PHDI. Saturated fat, total fat, cholesterol, monounsaturated fat, riboflavin, niacin, vitamin B5, and B12 were negatively associated with PHDI scores and emphasized that this is an expected result (12). Although there were relationships between some nutrients and PHDI scores in the current study, there was no relationship with all macro and micro nutrients as in the study by Cacau et al. (12). In their study, the mean PHDI scores were higher than our result. In this case, the generally low adherence of our population to PHDI might be reflected in the current result.

A study evaluating the effects of sustainable diets on healthy nutrition determined that daily dietary protein intake remained sufficient in high-income and middle-income countries, while it was below the recommended amounts in low-income countries (20). Intake of micronutrients increased, especially in high- and middle-income countries where significant amounts of animal-based foods were replaced with plant-based ones. When animal-based foods were replaced entirely with plant-based foods, baseline low levels of vitamin A, folate, iron, potassium, and fiber exceeded recommended values. However, calcium, pantothenate (vitamin B5), and vitamin B12 fell below recommended values in high-income and middle-income countries. In low-income countries, when a small amount of animal-based foods were replaced with plant-based foods, they were insufficient to increase potassium and vitamin A adequately, and riboflavin and calcium did not reach the recommended values (20). The associated financial burden is another crucial consideration when altering dietary habits to promote sustainability. In this regard, Hirvanen et al. noted that the EAT-Lancet estimates could not be met in low-income countries; for example, in South Asia, the reference diet will cost more than 1.5 times the average *per capita* household income per day. The authors also point out that fruit, vegetables, and animal products are among the most expensive food groups in the world (26). Currently, governmental initiatives to ensure food security are of utmost importance (27). Turkiye is in the upper middle-income country class (28). Our study did not find any statistical difference between the PHDI scores according to the working status (*p* > 0.05). However, the SHE Behaviors Scale score was higher in the working group. A study in India reported that even the wealthiest 5% of the population had unhealthy eating habits, low consumption of protein-rich food, fruits, and vegetables, and overconsumed processed foods (26). Concerning this, relevant government policies need to raise the public’s awareness about nutrition.

## 5. Limitations and strength

When evaluating the study findings, the following limitations must be considered. First, the research was conducted as a cross-sectional study in Erzurum, Turkiye (one of the metropolitan cities in the east of Turkiye). This cross-sectional study cannot determine a cause-and-effect relationship but evaluates the relationship between the measured variables. Second, nutritional habits differ between countries and even regionally. The study sample may not reflect Turkiye in terms of mean age, sex, and obesity prevalence. Consequently, it is essential to repeat the research in other regions/cities nationwide. Third, the participant’s weight and height were obtained from self-reports. Finally, the food consumption record could have been taken for at least three consecutive days instead of one. The strengths of the study are as follows: being an important research for evaluating the relationship between obesity and planetary health with a large sample size and being one of the first studies to evaluate individuals’ sustainable healthy eating behaviors, compliance of their diets with PHDI, and the factors affecting them in Turkish adults. Furthermore, to calculate the index, dietary intake was gathered using a 24-h recall, considered more accurate than a food frequency questionnaire.

## 6. Conclusion

The concept of a sustainable diet is relatively novel, and there are very few studies evaluating adherence to sustainable diets. To the best of our knowledge, this study is the first study to evaluate sustainable healthy eating behaviors of individuals, compliance of their diets with PHDI, and the factors affecting them in Turkish adults. This research showed that the participants’ PHDI index scores were low; therefore, the adherence to the EAT-Lancet recommendation was low. Assessment of participants’ diet quality will be beneficial in interpreting low adherence to PHDI. Low adherence to PHDI and low sustainable and healthy eating behaviors may be associated with obesity. Our findings indicate that education level can have a significant impact on sustainability. It is considered that sustainable and healthful nutrition education is necessary for environmental health sustainability. While adherence to a sustainable diet increases the intake of some nutrients, namely, those of animal origin, decrease. Clinical studies evaluating the effects of adherence to sustainable diets on adequate and balanced nutrition and health outcomes are recommended.

## Data availability statement

The raw data supporting the conclusions of this article will be made available by the authors, without undue reservation.

## Ethics statement

Ethical permission was obtained from the Erzurum Technical University Ethics Committee (Number of Meetings: 8, Decisions: 5, Date: 29.08.2022). The study was carried out in accordance with the principles outlined in the Helsinki Declaration. Informed consent was obtained from the participants. The studies were conducted in accordance with the local legislation and institutional requirements. The participants provided their written informed consent to participate in this study.

## Author contributions

MSM-Ç: conceptualization, methodology, formal analysis, writing–original draft, writing–review, and editing. OB: conceptualization, data curation, methodology, writing–original draft, writing–review, and editing. BK-B: conceptualization, data curation, methodology, writing–original draft, writing–review, and editing. EK: conceptualization, methodology, writing–review, and editing, and supervising. The authors have approved the final version submitted.

## Conflict of interest

The authors declare that the research was conducted in the absence of any commercial or financial relationships that could be construed as a potential conflict of interest.

## Publisher’s note

All claims expressed in this article are solely those of the authors and do not necessarily represent those of their affiliated organizations, or those of the publisher, the editors and the reviewers. Any product that may be evaluated in this article, or claim that may be made by its manufacturer, is not guaranteed or endorsed by the publisher.
