# Supplementary material for: Evaluation of sustainable and healthy eating behaviors and adherence to the planetary health diet index in Turkish adults: a cross-sectional study
Source: Front Nutr. 2023 Oct 2;10:1180880. doi: 10.3389/fnut.2023.1180880 (PMC10577287; doi:10.3389/fnut.2023.1180880)
Supplement: Supplementary file 1 [file Data_Sheet_1.docx]

**Supplementary File 1. Calculation of The Planetary Health Diet Index**

The PHDI is a diet index with 16 components divided into four groups: adequacy, optimum, ratio and moderation. The PHDI was calculated based on EAT Lancet Commission’s dietary recommendations (1). The PHDI uses a gradual scoring system as well as to being an energetic density index. Each component can receive a maximum of 10 or 5 points, resulting in a PHDI score between 0 and 150 (2). For the nuts and peanuts the cut off point is 11.6% and an intake equal to 0% was scored as 0 points, an between 0% to 11.6% was proportionally scored between 0 and 10 points. For the legumes, the cut off point is 11.3% and an intake equal to 0% was scored as 0 points and an intake from 0% to 11.3% was proportionally scored between 0 and 10. For the fruits, the cut off point is 5%, and consumption of 0% was scored with 0 points, while consumption between 0% to 5.0% was gradually scored from 0 to 10 points. For the vegetables, the cut off point is 3.1%, and consumption of 0% was scored with 0 points, while consumption between 0% to 3.1% was gradually scored from 0 to 10 points. For the whole grains, the cut off point is 32.4% and the consumption of 0% was scored as 0 points, while consumption between 0% to 32.4% was scored proportionally between 0 and 10. For the eggs, the average point is 0.8% and the upper limit is 1.5%. An intake from 0% to 0.8% was scored gradually from 0 to 10 points, while intake from 0.8% to 1.5% was scored inversely. An intake higher than 1.5% received 0 points. For the fish and seafood, the average point is 1.6% and the upper limit is 5.7%. An intake from 0% to 1.6% was scored gradually from 0 to 10 points, while intake from 1.6% to 5.7% was scored inversely. An intake higher than 5.7% get 0 points. For the tubers and potatoes, the average point is 1.6% and the upper limit is 3.1%. A consumption from 0% to 3.1% was scored gradually from 0 to 10 points, while consumption from 1.6% to 3.1% was scored inversely. An intake higher than 3.1% get 0 points. For the dairy group, an average point intake of 6.1% was accepted and 12.2% as upper limit. Consumption between 0% to 6.1% was scored gradually from 0 to 10 points, while between 6.1% to 12.2% was scored inversely. An intake higher than 12.2% get 0 points. For the dark green vegetables to total vegetables ratio the cut off value is 29.5% and for the red vegetables to total vegetables ratio is 38.5%, and values were scored gradually from 0 to 5 points. For the red meats, the cut off value is 2.4% and values between 0% and 2.4% scored gradually from 0 to 10 points. For the chicken and substitutes 5% was defined as upper limit and consumption above 5% scored 0 points, while zero intakes were given 10 points. An intake from 0% to 5.0% was scored inversely and gradually from 0 to 10 points. For the animal fats, 1.4% was cutf off point and consumption from 0% to 1.4% was scored inversely and gradually from 0 to 10 points. For the added sugars, the cut off value is 4.8% and intake below this lower limit received no points, while zero intakes received up to 10 points. Inversely and gradually from 0 to 10 points, an intake from 0% to 4.8% was scored (1, 2, 3).

**References**

1. Cacau LT, De Carli E, de Carvalho AM, Lotufo PA, Moreno LA, Bensenor IM, et al. Development and validation of an index based on EAT-Lancet recommendations: The Planetary Health Diet Index. Nutrients. 2021;13(5):1698.
2. Cacau LT, Benseñor IM, Goulart AC, Cardoso LO, Lotufo PA, Moreno LA, et al. Adherence to the planetary health diet index and obesity indicators in the Brazilian longitudinal study of adult health (ELSA-Brasil). Nutrients. 2021;13(11):3691.
3. Semba RD, de Pee S, Kim B, McKenzie S, Nachman K, Bloem MW. Adoption of the ‘planetary health diet’has different impacts on countries’ greenhouse gas emissions. Nature Food. 2020;1(8):481-4.
