# Supplementary material for: Evaluation of sustainable and healthy eating behaviors and adherence to the planetary health diet index in Turkish adults: a cross-sectional study
Source: Front Nutr. 2023 Oct 2;10:1180880. doi: 10.3389/fnut.2023.1180880 (PMC10577287; doi:10.3389/fnut.2023.1180880)
Supplement: Supplementary file 2 [file Data_Sheet_2.docx]

**Supplementary File 2.** Descriptive analysis of Planetary Health Diet Index components

|  | **Maximum score** | **Mean** | **SE** |
| --- | --- | --- | --- |
| **Red meat** | 10.00 | 4.55 | 0.25 |
| **Nuts and peanuts** | 10.00 | 2.2 | 0.30 |
| **Legumes** | 10.00 | 2.24 | 0.26 |
| **Chicken and substitutes** | 10.00 | 8.49 | 0.26 |
| **Fish and seafood** | 10.00 | 0.03 | 0.15 |
| **Eggs** | 10.00 | 0.01 | 0.13 |
| **Fruits** | 10.00 | 4.93 | 0.30 |
| **Vegetables** | 10.00 | 7.13 | 0.13 |
| **DGV/total ratio** | 5.0 | 0.12 | 0.20 |
| **ReV/total ratio** | 5.0 | 0.61 | 0.18 |
| **Whole cereals** | 10.00 | 0.38 | 0.32 |
| **Tubers** | 10.00 | 1.86 | 0.20 |
| **Dairy** | 10.00 | 3.1 | 0.33 |
| **Unsaturated oils** | 10.00 | 3.2 | 0.30 |
| **Animal fats** | 10.00 | 0.04 | 0.32 |
| **Added sugar** | 10.00 | 8.84 | 0.18 |

DGV: Dark green vegetetables/total ratio; ReV: Red vegetables/total ratio; SE: Standart Error

When calculating the PHDI components, chicken and substitutes, vegetables and added sugar were the top contributors. On the other hand, red meat and dairy components 14 reached around 30% of total components score (10 points). Fish and seafood, eggs, whole cereals and animal fats had the lowest scores.
